# Stimulation Modeling on Three-Dimensional Anisotropic Diffusion of MRI Tracer in the Brain Interstitial Space

**DOI:** 10.3389/fninf.2019.00006

**Published:** 2019-02-19

**Authors:** Wei Wang, Qingyuan He, Jin Hou, Dehua Chui, Mingyong Gao, Aibo Wang, Hongbin Han, Huipo Liu

**Affiliations:** ^1^Department of Radiology, Peking University Third Hospital, Beijing, China; ^2^Department of Radiology, The First People's Hospital of FoShan, Affiliated FoShan Hospital of Sun Yat-sen University, Foshan, China; ^3^Beijing Key Laboratory of Magnetic Resonance Imaging Devices and Technology, Peking University Third Hospital, Beijing, China; ^4^Department of Radiology, The Second Affiliated Hospital of Guangzhou Medical University, Guangzhou, Guangdong, China; ^5^Institute of Applied Physics and Computational Mathematics, Beijing, China

**Keywords:** interstitial space, anisotropic diffusion, mathematical model, brain, magnetic resonance, Gd-DTPA

## Abstract

**Purpose:** To build a mathematical model based magnetic resonance (MR) method to simulate drug anisotropic distribution *in vivo* in the interstitial space (ISS) of the brain.

**Materials and Methods:** An injection of signal intensity-related gadolinium-diethylenetriaminepentaacetic acid (Gd-DTPA), which is an exogenous drug, was administered, and its diffusion was traced in the ISS of the brain using MRI. Dynamic MRI scans were performed to monitor and record the changes in signal intensity in each pixel of the region of interest. The transport parameters were calculated using the modified equation to simulate three-dimensional anisotropic diffusion, which was resolved using a Laplace transform and a linear regressive model.

**Results:** After Gd-DTPA was introduced into the caudate nucleus, its distribution was demonstrated in real time. As the Gd-DTPA gradually cleared, the associated hyperintensity attenuated over time. The average diffusion coefficient (D) and the clearance rate constant (k) were (1.305 ± 0.364) × 10^−4^ mm^2^/s and (1.40 ± 0.206) × 10^−5^ s^−1^, respectively.

**Discussion:** The combination of trace-based MRI and modified diffusion mathematical models can visualize and measure the three-dimensional anisotropic distribution of drugs in the ISS of the brain.

## Introduction

Despite rapid progress in neuroscience, traditional oral or intravenous administration for brain diseases have consistently shown low efficiency (Fisher et al., 2009; Wolak and Thorne, 2013) and much more research needed to understand the brain activity underlying emotion, behavior, etc. (Yan et al., 2017a,b). Administering therapeutics through the interstitial space (ISS) of brain is considered a promising method of treating brain diseases based on the fluid dynamics of the interstitial fluid (ISF) in the ISS (N'djin et al., 2014; Lonser et al., 2015). This novel delivery strategy has demonstrated certain advantages compared with traditional drug delivery, including the ability to bypass the blood-brain barrier, wider targeted distributions throughout the brain volume, and reduced side effects (Xi et al., 2014). For example, the administration of a small dose cytidinediphosphate choline through the ISS of the brain demonstrated a greater efficiency for preventing trial ischemic stroke (Han et al., 2011). Despite its advantages, clinical therapies cannot be administered using this method until a greater understanding has been developed of the anatomy and physiology of the ISS as well as the regularity of drug distribution and clearance. So an appropriate mathematical model, which can stimulate drug distribution in ISS, is crucial to the emerging achievements and applications of the promising administration.

Based on recent anatomy development, brain is the particular example of porous media, where the ISS is the irregular, tortuous and narrow (mostly from 38 to 64 nm) space between neural cells and capillaries, and it occupies approximately 15–20% of the total brain volume and is filled with ISF (Sykova and Nicholson, 2008). Many important neural actives occur in the ISS, including neural cell communication, information processing and integration of coordinated responses to changes in the microenvironment (Xie et al., 2013; Kastellakis et al., 2015). It is believed that bulk flow and diffusion are the mechanisms underlying drug distribution in the ISS, which means that drugs can be driven by both the pressure gradient and the concentration gradient according to the fluid law of the ISF (Han et al., 2012). If the influence of pressure is neglected, diffusion is the sole factor in the distribution of drug ions in ISS. In a porous media several factors can impose constraints on the diffusion process. The primary factor is the geometrical structure and secondary are specialized features of medium. Substances contained in the ISF have a broad spectrum of physical and chemical differences and these differences influence the pH and viscosity of the ISF and impose constraints on the diffusion process (Shi et al., 2015). Currently, approaches for measuring the ISS *in vivo* primarily include radioactive tracers, real-time ion introduction, and integrated optical imaging. Based on Fick's second law of diffusion and the appropriate equation for ISS, these methods can measure the morphological parameters of the ISS in the local brain tissue (60~100 μm) (Sykova and Nicholson, 2008). However, they are sophisticated and cannot be used to monitor the drug distribution through the ISS of the brain due to their limitation of low image resolution and detection depth.

Tracer-based magnetic resonance imaging (MRI) technology employs gadolinium-diethylenetriaminepentaacetic acid (Gd-DTPA) to visual the transport procession the brain ISS, and the diffusion and clearance of the tracer over time at any pixel within the brain can be monitored and quantized (Kroenke and Neil, 2004; Han et al., 2014). The technique is based on the signal intensity increment (Δ*SI*) and its time course (Δ*SI*/Δ*t*), which can be used to obtain the rule for the tracer concentrations at any point within the brain over time (*C*/Δ*t*) (Xu et al., 2011). The technique demonstrates the anisotropic diffusion properties in brain ISS. Based on the isotropic diffusion equation applied in previous studies, a novel mathematical diffusion model was established for MR technique which can simulate the anisotropic diffusion process, and resolve the significant parameters of the diffusion and clearance process of Gd-DTPA in the brain ISS, including the diffusion coefficient (*D*) and the clearance rate constant (*k*).

## Materials and Methods

### MRI Protocols

A 3.0T MRI system (Magnetom Trio, Siemens Medical Solutions, Erlangen, Germany) with an eight-channel wrist coil was used with magnetization-prepared rapid acquisition gradient echo sequences (MPRAGE). The sequence parameters are as follows: repetition time = 1,500 ms, flip angle = 9°, field of view = 30 mm, slice thickness = 0.5 mm, resolution = 512 × 96, and voxel = 0.5 × 0.5 mm.

### Animal Models

The study was conducted in accordance with national guidelines, and the protocols were approved by the Ethics Committee of Peking University Health Center (Approval No. LA 2009-008). The experiments were performed on male Sprague Dawley rats weighing 280–360 g. Eight rats were anesthetized with an intraperitoneal injection of chloral hydrate (400 mg/kg) and then fixed in a stereotactic coordinate system (Lab Standard Stereotaxic-Single, Stoelting Co, Illinois, USA).Prior to injection, a MRI scan was performed to confirm the puncture position and obtain a basic reference image. A 2 μl dose of Gd-DTPA was slowly injected into the caudate nucleus of the brain at a rate of 0.2 μl/min according to pre-scan images. MRI scans were performed at 10, 30, 60, 90, 120, 180, 240, 300, and 360 min after injection.

**Figure 1:**
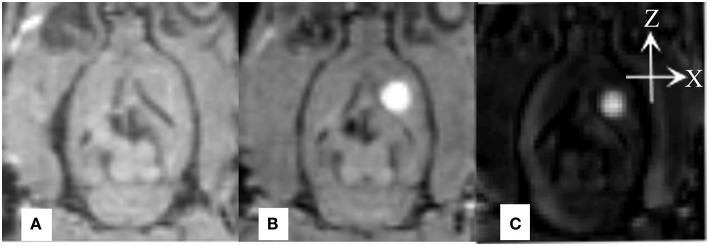
**(A)** Image of a rat brain prior to Gd-DTPA administration; **(B)** Image of a rat brain 10 min after administration. **(C)** Subtracted image. The data (Δ*SI*) for the tracer distribution along the X-axis and Z-axis can be obtained by calculating a series of rat brain images at different time points.

MATLAB-based software was developed to co-register the MR images of the same rat before and after the injection. The before-scanned images were then subtracted from the post-scanned images, and the signal intensity increment of the processed MR images was recorded using the associated software and denoted by Δ*SI*, which was used in the subsequent calculations (Figure 1). More detailed description on data processing method can be found in our prior paper (Han et al., 2014).

### Mathematical Model

For convenience, we assumed that the concentration at moment *t* and position be *C* = *C*(*x, y, z, t*). Drugs diffuse from areas of higher to lower concentrations. According to Fick's law, the amount of a drug that crosses the surface differential element Δ*SI* within time differential element Δ*t* is proportional to the normal differential quotient of the concentration along the surface, and the proportionality constant is the diffusion coefficient *D*. Conversely, an amount of the drug will be cleared through its combination with receptors, metabolism and entrance into brain cells. If the clearing rate is proportional to the concentration, then the proportional constant is the clearance rate constant *k* and self-secretion by nerve cells will result in an increase in endogenous drugs, such as dopamine in the case of Alzheimer's disease.

First, we selected a piecewise smooth and closed surface of the brain ISS and assumed that the space it encloses is Ω. From moment *t* to *t* + Δ*t*, Fick's law and the Gauss formula indicate that the quality of the drug that enters Ω by crossing surface *S* is as follows,

$$\begin{array}{l} M_{1}=\int_{t}^{t+\Delta t}∯{}_{S}\;(D_{x}\frac{\partial C}{\partial x}\cos\alpha +D_{y}\frac{\partial C}{\partial y}\cos\beta \\ \;+D_{z}\frac{\partial C}{\partial z}\cos\gamma )dSdt \end{array}$$

where cosα, cosβ, cosγ are the external normal cosine of, and *D*_*x*_*D*_*y*_*D*_*z*_ are the effective diffusion coefficients of the drugs in three orthotropic directions in the brain ISS. According to the Gauss formula,

$$\begin{array}{l} M_{1}=\int_{t}^{t+\Delta t}{∭}_{\Omega }(D_{x}\frac{\partial^{2}C}{\partial^{2}x}+D_{y}\frac{\partial^{2}C}{\partial^{2}y}\\ \;+D_{z}\frac{\partial^{2}C}{\partial^{2}z})dxdydzdt \end{array}$$

The loss quantity of drugs caused by clearance is as follows,

$$\begin{array}{l} M_{2}=\int_{t}^{t+\Delta t}{\int \int \int }_{\Omega }kCdxdydzdt \end{array}$$

Where *k* is the clearance constant. Suppose *Q* is a source. Volume fraction of ISS is denoted by α and may be formally defined as,

$$\begin{array}{l} \alpha =V_{ISS}/V_{Tissue} \end{array}$$

This term *Q* is divided by the volume fraction, reflecting the fact that molecules released into the ISS are restricted to a smaller volume than if they had access to the entire brain tissue. Then we have,

$$\begin{array}{l} M_{3}=\int_{t}^{t+\Delta t}{\int \int \int }_{\Omega }\frac{Q}{\alpha }dxdydzdt \end{array}$$

In addition, a change of the drug concentration is as follows,

$$\begin{array}{l} M_{4}={\int \int \int }_{\Omega }\left[C\left(x,y,z,t+\Delta t\right)-C\left(x,y,z,t\right)\right]dxdydz\\ \;=\int_{t}^{t+\Delta t}{\int \int \int }_{\Omega }\frac{\partial C}{\partial t}dxdydzdt \end{array}$$

According to the law of mass conservation,

$$\begin{array}{l} M_{4}=M_{1}-M_{2}+M_{3} \end{array}$$

Based on the arbitrariness of Δ*t*, the following is obtained,

(1) $$\begin{array}{r} \frac{\partial C}{\partial t}=D_{x}\frac{\partial^{2}C}{\partial x^{2}}+D_{y}\frac{\partial^{2}C}{\partial y^{2}}+D_{z}\frac{\partial^{2}C}{\partial z^{2}}-kC+\frac{Q}{\alpha } \end{array}$$

### Parameter Solution of Special Convection Diffusion Equations Based on the Exact Solution

By using Fourier Transform, we can obtain the exact solution of the three-dimensional convection diffusion Equation (1). However, the exact solution is very complex, and several special convection diffusion equations are applied in practical applications. Here we present two types of exact solutions for convection diffusion equations frequently used in health care. To simplify the problem, we assume that all coefficients of the equations are constants and provide special initial conditions and boundary conditions.

Instantaneous point-source convection diffusion model. For the convection diffusion Equation (1), the following initial and boundary conditions are given,

(2) $$\left\{ \begin{array}{r} C(x,y,z,t)=0,+\infty <x,y,z<-\infty ,t>0\\ \text{ ​}{C(x,y,z,)|}_{t=0}=0 \end{array}\right.$$

Where source term is instantaneous point-source presented as follows,

$$\begin{array}{l} Q\left(x,y,z,t\right)=M\delta \left(x\right)\delta \left(y\right)\delta \left(z\right)\delta \left(t\right) \end{array}$$

Where δ is the Dirac Function and *M* is the tracer mass, then the exact solution of the convection diffusion equation can be depicted as,

(3) $$\begin{array}{r} C\left(x,y,z,t\right)=\frac{M}{8\alpha {\left(\pi t\right)}^{3/2}\sqrt{D_{x}D_{y}D_{z}}}\\ \exp\left[-\frac{x^{2}}{4D_{x}t}-\frac{y^{2}}{4D_{y}t}-\frac{y^{2}}{4D_{z}t}\right]\exp\left(-kt\right) \end{array}$$

This model is easy to use medically in practice, for one simple instantaneous injection can satisfy all assumptions listed above.

### Continuous Point-Source Convection Diffusion Model

In the case of continuous injection, the concentration can be regarded as the integral of instantaneous point-source injection within a unit time over time domain,

(4) $$\begin{array}{r} C\left(x,y,z,t\right)=\int_{0}^{t}\frac{C_{0}q}{8\alpha {\left(\pi t\right)}^{3/2}\sqrt{D_{x}D_{y}D_{z}}}\\ \exp\left[-\frac{x^{2}}{4D_{x}t}-\frac{y^{2}}{4D_{y}t}-\frac{z^{2}}{4D_{z}t}\right]\exp\left(-kt\right)dt \end{array}$$

Where *C*_0_ is the concentration and *q* is the velocity of injection.

Several groups (more than five groups) of data, which includes tracer concentration distribution and the corresponding time and position, can be measured using MRI. The measured data are substituted into the exact solution of the above equation, parameters of *D*_*x*_,*D*_*y*_,*D*_*z*_,*k*,α can be attained using least square method. Then the average diffusion coefficient can be calculated as,

$$\begin{array}{l} D=\sqrt{D_{x}^{2}+D_{y}^{2}+D_{z}^{2}} \end{array}$$

## Results

**Figure 2:**
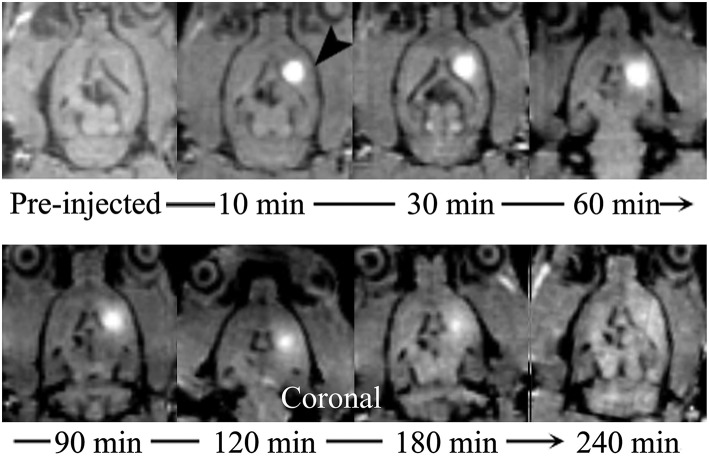
Coronal MR images demonstrate the process of diffusion and clearance of Gd-DTPA in the ISS of rat brain. After the Gd-DTPA tracer was injected into the ISS, the signal intensity of the caudate nucleus increased, and the hyper-intensity distributed around and the intensity attenuated gradually, which was related to the clearance of Gd-DTPA over time. Moreover, the anisotropic diffusion properties was demonstrated. Gd-DTPA was uniformly dissipated to the anterolateral frontal and temporal cortices and its distribution in posteromedial thalamus was not observed.

Real-time monitoring of the distribution of Gd-DTPA in the ISS revealed that after the Gd-DTPA tracer was injected into the ISS, signal intensity in the caudate nucleus increased. Gd-DTPA was uniformly dissipated to the anterolateral frontal and temporal cortices; no distribution in posteromedial thalamus was observed. The tracer was nearly cleared at 240 minutes, and an increased signal intensity was not demonstrated subsequently (Figure 2).

**Figure 3:**
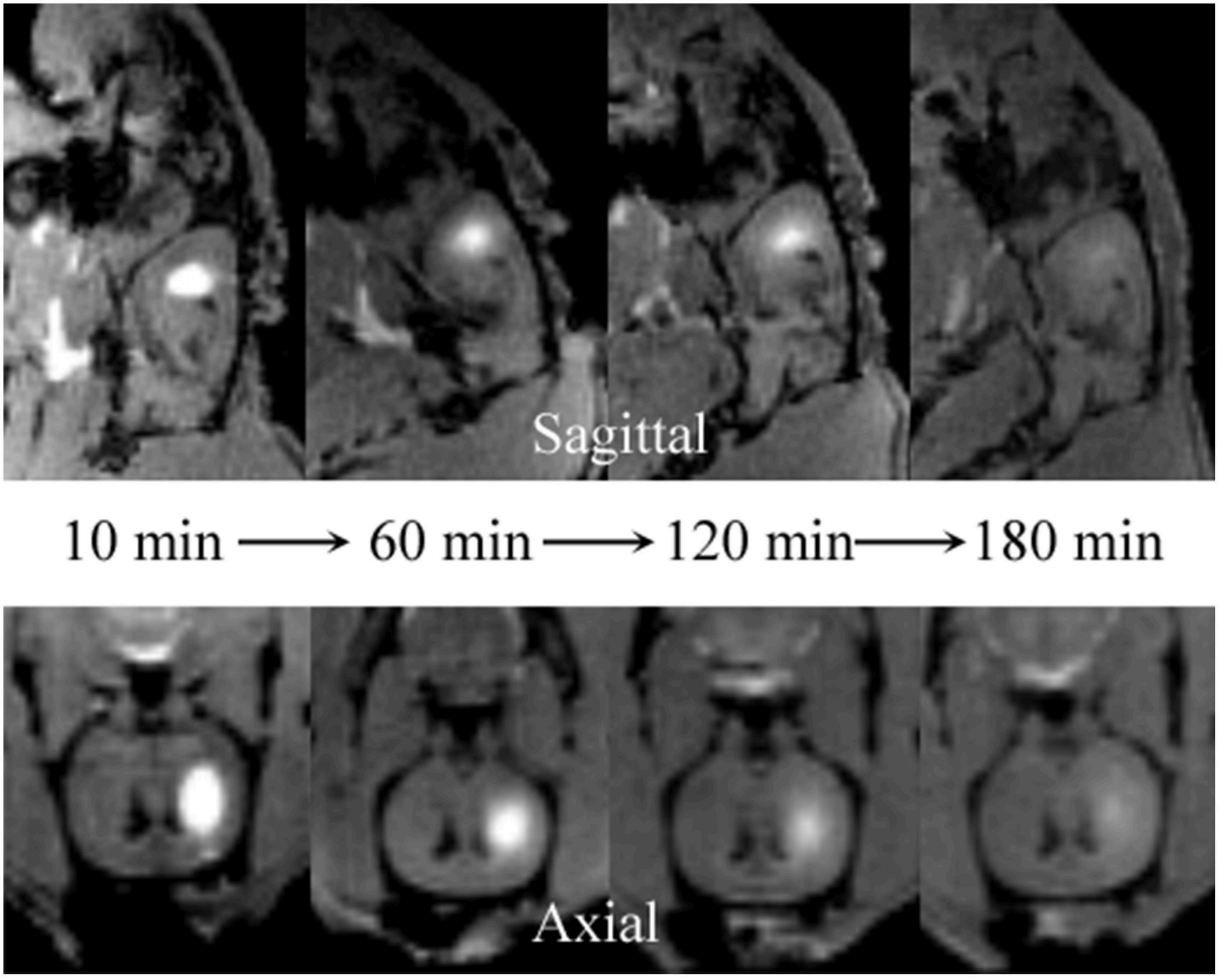
Distribution of Gd-DTPA demonstrated in real time in ISS of rat brains using multi-view images. The sagittal **(upper)** images demonstrate the process of Gd-DTPA anterior and ventral-dorsal diffusion. The axial **(lower)** demonstrate the lateral and ventral-dorsal Gd-DTPA diffusion.

Among the currently available brain ISS measurement techniques, the tracer-based MRI technique is unique in producing 3-D images of substance distributions in ISS. The images can demonstrate the diffusion progression in arbitrary directions (Figure 3).

**Figure 4:**
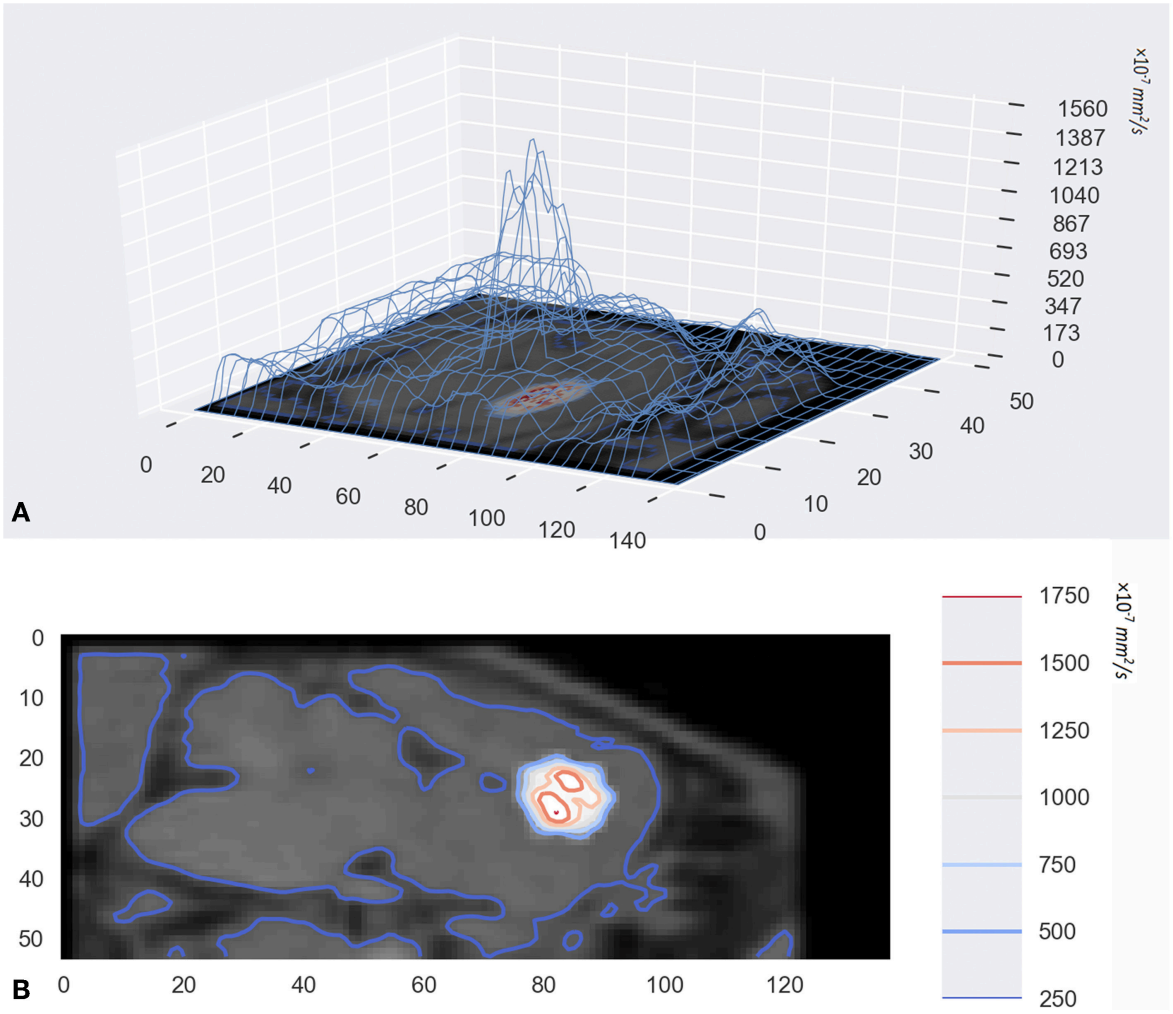
**(A)** was the 3D wireframe and the height of the contour line represents the level of D. Panel **(B)** was a contour map superimposed on the original MR image. They were drawn with a Python package named Matplotlib.

In addition to visualization of diffusion progression in ISS, the tracer-based MRI technique provides quantitative measures of the distribution rate (Figure 4). Observed drug concentrations at different times and locations gave parameter estimates average *D* = 1.305 ± 0.364 × 10^−4^ and *k* = 1.40 ± 0.206 × 10^−5^ *s*^−1^.

A comparison of the novel tracer-based MRI and classical approaches is provided in Table 1.

**Table 1:** The comparison of the novel tracer-based MRI and classical approaches.

	**Tracer-based MRI**	**Real-time ion introduction**	**Integrative optical imaging**
Tracer	Gd-DTPA	Positive ion (TMA^+^)	Fluorescent probe (Dextron)
Signal	Radio wave	Electric potential	Fluorescence
Detection Capability	Global	Distance < 200 μm	Depth < 200 μm
Imaging	3D	No	2D
Parameters	*D*, λ, t_1/2_, *k*	D, λ, α	D, λ
Influenced by brain activity	No	Yes	No
Mathematical model	3D anisotropic diffusion equation	1D Isotropic diffusion equation	2D Isotropic diffusion equation

*TMA^+^: cation tetramethylammonium*.

*D: diffusion coefficient*.

*λ tortuosity*.

*α volume fraction*.

*t_1/2_ half-life*.

*k clearance rate constant*.

## Discussion

In our research, drug diffusion and clearance in the ISS of the brain can be monitored in real time using multi-view MR images. Superiority in the safety, enhanced soft-tissue contrast and global imaging advantages, MRI is an excellent technique for the *in vivo* imaging of biological tissues (Hosseini et al., 2016; Chen et al., 2018; Yan et al., 2018).

Among the currently available ISS measurement techniques, MRI is unique in that it can detect the ISS in the deep regions at the whole-brain scale (Lei et al., 2017). Gd-DTPA is the preferred contrast-drug in clinic applications and has been used to trace substance transport in the ISS in previous studies (Benjaminsen et al., 2008). Gd-DTPA can shorten the spin-lattice relaxation time of the hydrogen nuclei in water molecules within an effective distance of 2.5 angstroms and highlight endogenous water molecules in T1-weighted MR images (Patil and Johnson, 2011). After the drug is injected into the ISS, it will diffuse and be cleared, which presents as the attenuation of signal intensity in a series of MR images. MPRAGE sequence was used to acquire a series of 3D MR images. Additionally, a MATLAB-based software was developed to post-process these images, including the removal of MRI noise and the rigid registration and conversion of MR signal intensity increment to Gd-DTPA concentrations. The technique, which can be referred to as a tracer-based MRI, can image the distribution and clearance of Gd-DTPA in the ISS and provide mathematical models by recording the varying drug concentrations with respect to time with the goal of estimating the drug diffusion and clearance parameters, including the effective diffusion coefficient *D* and the clearance rate constant *k*.

The effective diffusion coefficient is defined as the diffusion scope of the substances through a medium in unit time, where the unit is. This measure reflects the rate of diffusion in the medium and is affected by various factors, such as the ISS structure, dead space, cell matrix-induced blockages, negative ions attached to molecules and drug characteristics, which is the most intuitive parameter for indicating the drug diffusion scope in the brain (Nicholson, 2005). By using different methods or tracers, the measurements of the effective diffusion coefficient of substances in the ISS varied from 0.38 to 20 × 10^−4*m*^*m*^^2^/*s*^ (Sykova and Nicholson, 2008).

The clearance rate *k* indicates the loss of the drug from the brain ISS and includes the amount of the drug that enters the blood-brain barrier, combines with receptors, enters cells and drains from the brain ISS. The rate of drug drainage in the brain ISS has the greatest effect on the *k* value. According to Nicholson's research, the main methods by which drugs drain from the brain include blood circulation, lymph circulation and cerebrospinal fluid circulation (Iliff et al., 2013; Louveau et al., 2015). Therefore, to maintain an effective drug concentration level to cure diseases, the *k*-value must be accurately estimated.

This research establishes modified mathematical models using Cartesian and spherical coordinates that simulate the anisotropic diffusion process using numerical differentiation and a linear regressive method. There are several key points related to the approach that should be discussed. Firstly, the effective diffusion coefficient and the clearance rate *k* are affected by the characteristics of the administered drug, such as the structure, size and polarity of the drug molecule. Therefore, to obtain these parameters for certain drugs using animal models, it is essential to inject a MR tracer, such as Gd-DTPA, to mark the drugs. After acquiring the data, our model will estimate the corresponding parameters. Secondly, drug administration in the proposed model is performed through bolus injection rather than by continuous administration, and the concentration gradient becomes the sole driving force and diffusion dominates. Mériaux recently announced an interesting result that 7T MRI, combined with a non-invasive probe delivery technique, could be used to estimate the tortuosity values in deep tissue regions *in vivo* with excellent sensitivity, together with spatial and temporal resolutions (Mériaux et al., 2018). However, even if imaged by the most advanced MR, nanoscale ECS cannot be addressed directly at present. In our opinion, it is a complex and time-consuming project for ECS research, that involves MR protocol and probe, signal detection and processing, mathematical models, test, and trial. Developing appropriate mathematical model and signal processing should be its key highlights. Drug delivery by ECS is the most promising field in the research on ECS, and a platform using 1.5T or 3T MRI can be developed more easily than one using 7T MRI. Lastly, thanks to that the tracer Gd-DTPA is not self-secreted in the brain, the parameter value that reflects the endogenous drug dose change is zero, and the drug dose presents a continuous decline to a stable state at a concentration of zero.

## Author Contributions

HH contributed conception and design of the study. HL constructed the mathematical model. JH performed the data collection. WW, QH, and AW wrote the first draft of the manuscript. DC and MG wrote sections of the manuscript. All authors contributed to manuscript revision, read and approved the submitted version.

### Conflict of Interest Statement

The authors declare that the research was conducted in the absence of any commercial or financial relationships that could be construed as a potential conflict of interest.
